# *ABCB1* polymorphisms are associated with clinical response to nabiximols in patients with multiple sclerosis-related spasticity

**DOI:** 10.1186/s42238-025-00333-4

**Published:** 2025-09-29

**Authors:** Alessandra Gemma, Marco Mauri, Paola Banfi, Maurizio Versino, Alen Zollo, Filippo Martinelli Boneschi, Franca Marino, Marco Cosentino, Marco Ferrari

**Affiliations:** 1 Center for Research in Medical Pharmacology, University of Insubria, Via Monte Generoso n.71, Varese, 21100 Italy; 2 Neurology and Stroke Unit, ASST Sette Laghi Hospital, Varese, 21100 Italy; 3 Department of Biotechnology and Life Sciences, University of Insubria, Varese, 21100 Italy; 4 Center for Neurotechnology and Experimental Brain Therapeutics, Department of Health Sciences, University of Milan, Milan, Italy; 5 Clinical Neurology Unit, Department of Health Sciences, Azienda Socio-Sanitaria Territoriale Santi Paolo e Carlo, University of Milan, Milan, Italy

**Keywords:** Multiple sclerosis, Single nucleotide polymorphisms, Nabiximols, Spasticity, ABCB1

## Introduction

Multiple Sclerosis (MS) is a chronic inflammatory, demyelinating, and neurodegenerative disease affecting the central nervous system (Filippi et al. 2018). MS patients experience a wide range of symptoms, with spasticity affecting at least two-thirds of individuals (Kister et al. 2013). Nabiximols, NBX oromucosal spray (Sativex^®^), drug containing Tetrahydrocannabinol (THC) and cannabidiol (CBD), is approved for adult patients with moderate-to-severe MS-associated spasticity (Kleiner et al. 2023; Chan et al., 2022; eMC 2022).

Despite NBX efficacy, only 60–70% of MS patients respond to treatment with this drug (Almog et al. 2020), and up to now there are no reliable predictors its efficacy that could be useful in clinical practice.

Genetic factors represent crucial contributors to variability in drug. In particular, single nucleotide polymorphisms (SNPs) may predict drug, efficacy and safety in many clinical settings (Reviewed in Owusu Obeng et al. 2021).

Both THC and CBD act on cannabinoid receptors (CBR) 1 and 2, which are encoded by the cannabinoid receptor genes (*CNR*) 1 and 2 (Martini et al. 2023). SNPs in *CNR1*, such as rs1049353 and rs2023239 and in *CNR2* (rs2501431), influence various receptor functions, including depression (Mitjans et al. 2012), happiness perception (Matsunaga et al. 2014) and childhood obesity (Col Araz et al. 2012). THC and CBD are substrates for P-glycoprotein (P-gp), an efflux pump encoded by the ATP Binding Cassette Subfamily B Member 1 (*ABCB1*) gene (Martini et al. 2023). SNPs in the *ABCB1*, such as rs1128503 and rs1045642, have been associated with interindividual variability in drug response (Brambila-Tapia 2013), including response to cannabinoids (Babayeva et al., 2023). These polymorphisms have also been proposed as potential predictors of individual responses to analgesics, including opioids (Fernandez et al., 2012) and cannabis-based treatments (Poli et al. 2022).

Additionally, THC and CBD are metabolized by the CYP450 enzyme family, by CYP2C9, CYP2C19, and CYP3A4 (Martrini at al., 2023; Babayeva et al., 2023), and it has been shown that SNPs, such as rs1799853 (CYP2C9) and rs4244285 (CYP2C19), dramatically reduce metabolic activity of these enzymes (Zanger et al. 2008).

In the present study, we investigated the relationship between all abovementioned SNPs and response to drug treatment in a cohort of MS patients in which MS-related spasticity was treated with NBX.

## Methods

### Patients

This exploratory genetic study retrospectively enrolled all patients diagnosed with MS based on the McDonald criteria 2017 and treated with NBX for MS-related spasticity at the “Centro Sclerosi Multipla, Ambulatorio Malattie Demielinizzanti - Ospedale di Circolo e Fondazione Macchi, Varese, Italy” from May 2014 to June 2024. Exclusion criteria were: (i) use of other spasticity-related pain medications, such as anticonvulsants/antiepileptic, antidepressants, or opioids, (ii) previous exposure to NBX or other cannabinoids; (iii) presence of kidney or liver diseases; (iv) concurrent treatments with drugs known to affect NBX metabolism or transport; (v) other conditions/disease causing chronic pain (e.g., cancer, trigeminal neuralgia, fibromyalgia, diabetes, arthritis).

Response to NBX was evaluated using the Patient-rated spasticity 0–10 Numeric Rating Scale (NRS) as well as through clinical evaluation (Dworkin et al. 2005; Ferré et al., 2016). According to the current accepted definition, a reduction of ≥ 20% from baseline on the NRS after 4 weeks of treatment represents a minimal clinically difference, while a reduction of ≥ 30% indicates a clinically important difference (Dworkin et al. 2005; Ferré et al., 2016).

On this basis, we decided to consider patients who achieved at least a 30% reduction in NRS at week 4 compared to baseline and continued with NBX treatment as responders.

Patients who did not achieve a 30% reduction in NRS scores within the fourth week of treatment and were switched to alternative spasticity therapy were considered as non-responders.

### SNPs selection criteria and genotyping

The SNPs were selected based on their frequency in the Caucasian population, which must be greater than 10%, and the availability of information regarding their biological and clinical effects.

Drawing from our previous experience (Ferrari et al. 2016, 2024; Comi et al. 2017), the choice of SNPs with high frequencies increases the probability of identifying potential differences in their frequency in relation to relevant phenotypic aspects, even within a relatively small patient cohort. Furthermore, selecting SNPs with well-established biological effects enhances the likelihood of their contribution to determining patients’ phenotype. A detailed description of SNPs included in the study are reported in Table 1.

**Table 1:** SNPs included in the study

Gene	Variant	*N*.C.	A.F. (%)	Biological effect
*CNR1*	rs1049353	1359G > A	27	Associated to addiction (Hryhorowicz, 2018) and happiness (Matsunaga, 2014).
	rs2023239	-3163 A > G	17	Major risk of adverse effects (Ishiguro, 2007).
*CNR2*	rs2501431	24201643G > A	58	Major risk of depression (Mitjans, 2012).
*ABCB1*	rs1128503	1236 C > T	43	Lower expression (Wang and Sadée 2006).
	rs1045642	3435 A > T	52	Lower expression (Wang and Sadée 2006; Hoffmeyer, 2000).
*CYP2C9*	rs1799853	9133 C > T	12	Reduced activity (Zanger et al. 2008)
*CYP2C19*	rs4244285	681G > A	15	No activity (Zanger et al. 2008).

Abbreviations: N.C., nucleotide change; A.F., allelic frequency in Caucasian population; *ABCB1*, ATP Binding Cassette subfamily B member 1; *CYP*, CYtochrome P450; *CNR1*, CaNnabinoid Receptor 1 gene; *CNR2*, CaNnabinoid Receptor 2 gene

DNA was extracted using FTA Elute Cards (GE Healthcare Bio-Sciences AB, SE-751 84 Uppsala, Sweden) according to the manufacturer’s instructions (https://it.vwr.com/store/product/7997552/fta-elute-cards-whatmantm).

Selected SNPs were genotyped using a Real-Time PCR system (StepOne^®^, Thermo Fisher Scientific, Waltham, MA, USA) with a pre-designed TaqMan^®^ genotyping assay (Thermo Fisher Scientific, Waltham, MA, USA).

### Statistical analysis

Data are shown as the mean ± standard deviation (SD), unless otherwise stated. The statistical significance of the differences between groups was assessed by the Mann–Whitney U-test or by One-way analysis of variance followed by Bonferroni’s Multiple Comparison Test as appropriate. The evaluation of Hardy-Weinberg equilibrium was assessed using the χ2-test (*P* < 0.05). Differences in allele frequencies between groups were analyzed by the χ2-test for trend or the Fisher’s exact test (recessive model). The odds ratio (OR) with a 95% confidence interval (CI) was calculated using a recessive model (wild type/heterozygous vs. homozygous for SNP).

Statistical analyses were performed using GraphPad Prism version 5.00 for Windows (GraphPad Software, San Diego, California, USA, www.graphpad.com).

## Results

### Patients

From clinical records, we identified 47 patients treated with NBX for MS-related spasticity from May 2014 to June 2024. Of these, one patient was excluded due to concomitant opioid treatment for cancer-associated pain, and another was excluded for using carbamazepine to manage trigeminal neuralgia-related pain. Table 2 shows demographic and clinical characteristics of the 45 patients finally enrolled.

Among the enrolled patients, 29 achieved an NRS score reduction of 30% or greater after 4 weeks of NBX treatment and were included in the responder group. For 16 patients, the NRS score reduction did not reach 30%. These patients switched to alternative pain treatments and were included in the non-responder group.

We did not find any difference between groups in terms of gender, age, MS type, disease duration, MS therapy, Multiple Sclerosis Severity Score (MSSS), Expanded Disability Status Scale (EDSS), or NBX dosage (Table 2).

**Table 2:** Clinical and demographic characteristics of MS patients. * = *P* < 0.001 vs. non-responders

	All	Responders	Non-responders
Number of subjects	45	29	16
Gender (male/female)	18/27	14/15	4/12
Age (years, mean ± SD)	53.0 ± 11.1	53.1 ± 12.2	52.9 ± 9.2
Disease duration (years, mean ± SD)	17.3 ± 10.1	16.3 ± 10.6	19.0 ± 9.2
EDSS (mean ± SD)	5.6 ± 1.8	5.4 ± 1.8	5.9 ± 1.6
MSSS (mean ± SD)	6.2 ± 2.1	6.1 ± 2.2	6.2 ± 2.0
**MS type**			
Relapsing Remitting	25	19	6
Primary Progressive	11	6	5
Secondary Progressive	9	4	5
**MS therapy**			
Ocrelizumab	13	7	6
Natalizumab	4	3	1
Teriflunomide	4	3	1
Dimethyl fumarate	2	2	0
Fingolimod	6	3	3
No therapy	15	10	5
**NBX dosage** (puffs/ day, mean ± SD)*	5.6 ± 2.5	5.8 ± 1.8	5.4 ± 3.0
**NRS score**			
before NBX (mean ± SD)	7.1 ± 1.2	7.1 ± 1.4	7.1 ± 1.2
after NBX (mean ± SD)	4.1 ± 2.1	2.6 ± 1.8*	5.9 ± 0.8
% reduction (mean ± SD)	47.0 ± 28.5	64.7 ± 21.1*	14.7 ± 2.0

Abbreviations: EDSS, Expanded Disability Status Scale; MSSS, Multiple Sclerosis severity scale; MS, multiple sclerosis; THC, Δ9-tetrahydrocannabinol; CBD, cannabidiol; NRS, numerical rating scale. *, 1 puff NBX = 100 µl spray including 2.7 mg THC and 2.5 mg CBD

### Correlation between patient genotype and response to NBX

All SNPs were in Hardy–Weinberg equilibrium (data not shown). Among the 29 patients who responded to NBX therapy, 18 (62%) were carriers of the C allele in the rs1128503 SNP in *ABCB1*, and 15 (52%) were carriers of the A allele in the rs1045642 SNP in *ABCB1*, while 11 (38%) and 14 (48%), respectively, were homozygous for the T allele. None of the 16 patients who did not respond to NBX therapy were homozygous of the T allele (Table 3). Using a χ2 test for trend, we found that the frequency of the T allele in both rs1128503 (1236 C > T) and rs1045642 (3435 A > T) in *ABCB1* was significantly higher in responders compared to non-responders (*P* < 0.0010 and *P* < 0.0012 respectively). This result was confirmed by the Fisher exact test. The odds ratio (O.R.) for response to NBX was 20.5 (95% C.I.: 1.1–376.1; = 0.0039) for rs1128503, and 30.9 (95% C.I.: 1.7–563.2; *P* = 0.0006) for rs1045642. SNPs in *CNR1*, *CNR2*, *CYP2C9*, and *CYP2C19* were not significantly associated with the response to NBX (Table 3).

**Table 3:** Correlations between patient’s genotype and NBX response. * = χ2-test for trend; # = Fisher exact test

Gene	SNP	Genotype	Responder *n*. (%)	Non-responder *n*. (%)	P*	*P*#	Odds ratio (95% C.I.)
*CNR1*	rs1049353	G/G G/A A/A	21 (72.4) 7 (24.1) 1 (3.5)	8 (50.0) 7 (43.7) 1 (6.3)	0.1580	1.000	1.9 (0.1–32.0)
	rs2023239	A/A A/G G/G	24 (82.8) 5 (17.2) 0 (0.0)	9 (56.2) 5 (31.3) 2 (12.5)	0.0629	0.1212	10.2 (0.5-226.1)
*CNR2*	rs2501431	G/G G/A A/A	3 (10.3) 18 (62.1) 8 (27.6)	2 (12.5) 9 (56.2) 5 (31.3)	0.9364	1.000	1.2 (0.3–4.5)
*ABCB1*	rs1128503	C/C C/T T/T	1 (3.4) 17 (58.6) 11 (38.0)	4 (25) 12 (75.0) 0 (0.0)	0.0010	0.0039	20.5 (1.1-376.1)
	rs1045642	A/A A/T T/T	3 (10.3) 12 (41.4) 14 (48.3)	5 (31.3) 11 (68.7) 0 (0.0)	0.0012	0.0006	30.9 (1.7-563.2)
*CYP2C9*	rs1799853	C/C C/T T/T	24 (82.8) 4 (13.7) 1 (3.5)	11 (68.7) 5 (31.3) 0 (0.0)	0.4787	1.000	0.6 (0.02-15.0)
*CYP2C19*	rs4244285	G/G G/A A/A	19 (65.5) 8 (27.6) 2 (6.9)	10 (62.5) 6 (37.5) 0 (0.0)	0.4858	0.5313	1.140 (0.01–7.4)

Abbreviations: *CNR1*, CaNnabinoid Receptor 1 gene; *CNR2*, CaNnabinoid Receptor 2 gene; *ABCB1*, ATP Binding Cassette subfamily B member 1 gene; CYP, CYtochrome P450

**Fig. 1:**
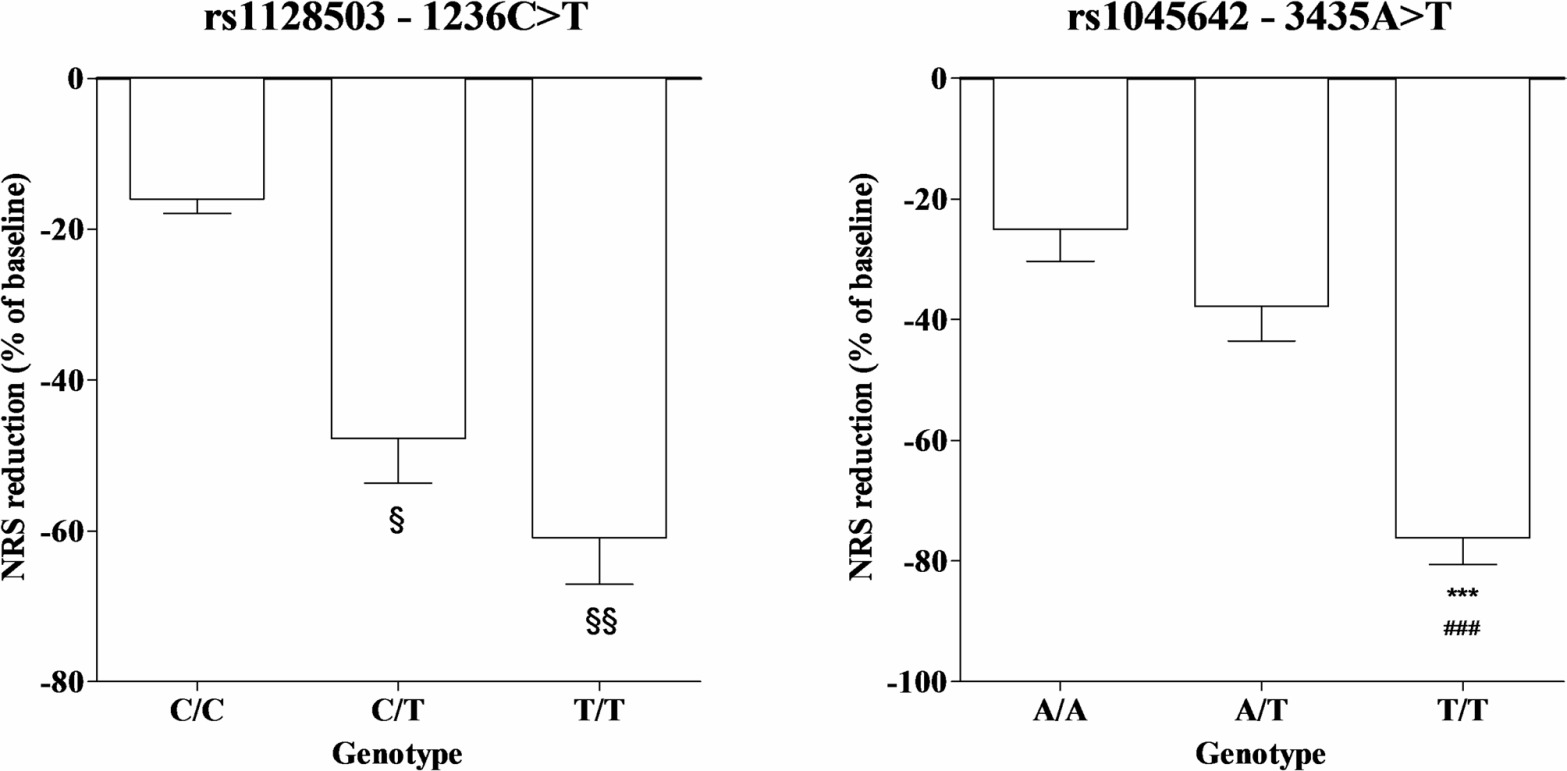
Relationship between SNPs *ABCB1* and % of reduction in NRS score. § = *P* < 0.05 C/T vs. C/C; §§ = *P* < 0.01 T/T vs. C/C; *** = *P* < 0.0001 T/T vs. A/A; ### = *P* < 0.0001 T/T vs. A/T

Subjects with the T/T and C/T genotypes for the rs1128503 SNP in *ABCB1* exhibited a significantly greater percentage reduction in NRS scores following NBX treatment compared to those with the ancestral C/C genotype (*P* < 0.05 and *P* < 0.001 respectively). Similarly, patients with the T/T genotype for the rs1045642 SNP showed a significantly greater percentage reduction in NRS scores compared to both the C/C and C/T genotypes (*P* < 0.0001) (Fig. 1). SNPs in the *CNR* and CYP did not show any notable association with NRS scores (**data did not show**).

## Discussion

The main finding of this study is that the T allele in the *ABCB1* rs1128503 (1236 C > T) and rs1045642 (3435 A > T) is predictive of response to NBX treatment in MS-related spasticity. Specifically, the T allele, previously associated with reduced P-gp efflux activity (Wang and Sadée 2006), was more frequent among treatment responders. Moreover, patients carrying the T allele in either SNPs showed significantly greater percentage reductions in NRS scores following NBX treatment compared to those with ancestral or heterozygous genotypes.

Several studies have demonstrated the efficacy of cannabinoids, including NBX, in management of clinical conditions including cancer pain, epilepsy and some inflammatory disease. However, it is well established that, despite their therapeutic benefits, individual response to cannabinoids as well as side effect drug induced is influenced by patient’s genetic background (reviewed in Jose et al. 2020). To date, only a limited number of studies have evaluated the role of SNPs in *ABCB1* in response to cannabinoid treatments. Among these, Poli and colleagues (2022) demonstrated that haplotypes containing the rs1045642 SNP may influence the effectiveness of cannabis in managing chronic pain, as well as its associated side effects (Poli et al. 2022). Beyond *ABCB1* SNPs, other SNPs have also been linked to cannabinoid response. For Example, the CYP2C9*2 and CYP2C9*3 SNPs were associated with higher plasma levels of CBD (Batinic et al. 2023) and THC-induced side effects (Wolowich et al. 2019). Moreover, in epilepsy patients, SNPs in *ABC* family, were found to enhance CBD response (Davis et al., 2021). Finally, SNPs in the *CNR1* have been shown to affect THC’s impact in patients with irritable bowel syndrome (Wong et al. 2012).

In light of these findings, several factors, including SNPs in *ABCB1*, *CYP2C9*, and *CNR1*, have been proposed as potential determinants of cannabinoid-related dependence and side effects (Kitdumrongthum and Trachootham 2023). However, reliable indicators to predict the efficacy of NBX in clinical practice have to be identified yet.

In this study, we found, for the first time, that patients carrying the T allele in both rs1128503 and rs1045642 SNPs exhibited a better response to NBX. Our working hypothesis is that higher frequencies of SNPs known to reduce P-gp activity may increase NBX bioavailability and facilitate its passage across the blood-brain barrier. This could result in higher drug concentrations at its site of action, thereby improving NBX efficacy. Although our findings are hypothesis-generating, they hold promise for advancing precision medicine by helping to identify patients most likely to benefit from NBX treatment, potentially improving its efficacy by enhancing its presence at the site of action. However, further validation in larger patient cohorts is necessary before this method can be considered a reliable tool for predicting patient response to NBX therapy in clinical settings.

In this study, no correlation was found between NBX response and SNPs in either CNR1 or CNR2. One possible explanation is that, while SNPs in ABCB1 may enhance THC/CBD penetration across the blood-brain barrier, hereby amplifying the therapeutic effect, SNPs in CNR1 and CNR2 may not produce functional changes sufficient to influence NBX efficacy at standard doses. It is also important to consider that, although CB1 and CB2 are the primary cannabinoid receptors, cannabinoids can modulate additional pathways, including TRP channels, 5-HT₁A receptors, and PPARγ (Furgiuele et al. 2021). Therefore, it cannot be excluded that the effects of SNPs in *CNR* are offset by these alternative targets, potentially neutralizing the impact of individual receptor variants (De Petrocellis and Di Marzo 2010).

Similarly, no association was observed between SNPs in CYP2C9 and CYP2C19 and NBX response. However, only the most common variants were analyzed, and it remains possible that these SNPs may affect THC/CBD metabolism primarily in cases of high-penetrance heterozygosity or homozygosity. Additionally, rare variants, specific haplotypes, or SNPs in other enzymes with a lesser role in THC/CBD metabolism, not included in this study, could also influence cannabinoid metabolism and, consequently, NBX efficacy.

We acknowledge that our study has some limitations, primarily the retrospective design and the small sample size. However, it must be considered that this is an exploratory study with strict inclusion criteria (i.e. Analgesic monotherapy with NBX, exclusion of concomitant treatments with drugs influencing NBX metabolism/transport, and/or diseases causing chronic pain). The strict inclusion criteria reduce possible confounders and, in turn, increase the likelihood of evaluating the role of genetics in response to NBX treatment. Moreover, although the exploratory nature of our study did not require a formal sample size calculation (Kimmelman et al. 2014), we performed a preliminary assessment of the sample size based on our study design parameters, the genotype distribution, and the NBX efficacy reported in the literature, using the Kane SP Sample Size Calculator (ClinCalc; https://clincalc.com/stats/samplesize.aspx). Assuming a minimum allele frequency of 10% for the rarest SNP, a 30% non-responder rate (Almog et al. 2020), and a risk ratio of 2.5 (moderate association), a sample of 45 participants would provide 80% power to detect a gene–treatment interaction at an alpha level of 5% (see also Chi-hong Tseng & Yongzhao Shao, 2010).

In conclusion, in this exploratory study, we have shown, for the first time, a relationship between a patient’s genetic profile and response to NBX treatment. If confirmed in a prospective study involving a larger cohort of patients, our results could pave the way for the identification of new, useful tools for predicting the response to NBX treatment in MS patients suffering from spasticity, ultimately allowing for personalized therapy in patients with indications for this drug.

## Data Availability

The data that support the findings of this study are available from the corresponding author upon reasonable request. Data are in controlled access data storage at Centre for Research in Medical Pharmacology, University of Insubria, Varese, Italy.

## Declarations

### Additional information

The Authors wish to dedicate this study to the loving memory of Paola Banfi, dearest friend, caring physician, and invaluable scientist.
